# PD24 - Oral food challenges to peanut in the pediatric population at the Allergy Department of the University Hospital of Montpellier, France

**DOI:** 10.1186/2045-7022-4-S1-P24

**Published:** 2014-02-28

**Authors:** Maria Chiara Leoni, Marie Pascale Demoly, Anca Mirela Chiriac, Gian Luigi Marseglia, Pascal Demoly

**Affiliations:** 1 Dipartimento di Scienze Clinico-Chirurgiche, Diagnostiche e Pediatriche, Ambulatorio di Immuno-Pneumo-Allergologia Pediatrica, Fondazione IRCCS Policlinico San Matteo, University Hospital of Pavia, Pavia, Italy; 2 Département de Pneumologie et Addictologie, Unité d’Allergologie INSERM U657, HÔpital Arnaud de Villeneuve, University Hospital of Montpellier, Montpellier, France

## Objective

We describe the experience of our department with positive oral food challenges (OFC) to peanut in children, over a 10-year period.

## Methods

Children who underwent a positive OFC to peanut between November 2001 and November 2012 in the Allergy Department of the University Hospital in Montpellier, France, were analysed with respect to demographics, history of atopic disease, specific IgE values, skin tests results and the severity of both the index reaction and the one elicited by the OFC (according to the Sampson grades of severity)[1].

## Results

69 OFC to peanut were positive in 59 (38 male and 21 female) patients, aged 3-16 years (mean age, 7.56 years). 129 OFC turned out negative over the same period of time. All the patients were atopic. The most common allergens were: grass mix (44.1%), cat and dog dander (42.6%), house dust mites (38.2%), molds (29.4%) and cypress pollen (20.5%). 24 (35.2%) patients had allergic rhinitis and 45 (66.2%) had asthma (n=26 GINA 1, n=18 GINA 2, n=1 GINA 3). 29 (42.6% ) patients suffered from atopic eczema. 26 patients (41%) had no history of previous ingestion of peanuts and the avoidance regimen had been recommended either because of positive IgE and/or skin tests (in the context of atopic disease in early life) or because of reactions to cross-reactive tree nuts. 32 OFC were conducted in this population, eliciting 20 grade I reactions (62.5%), 11 (34.4%) grade II reactions and one anaphylactic shock. In the other patient group, with a clinical history of reaction to peanut itself, 29 patients (49.2%) had a history of a grade I reaction and 4 (6.7%) described grade II reactions. The 37 OFC performed in these patients were followed either by grade I or II reactions, namely the reaction was just as severe in 21 (56.7%), more severe in 13 (35.1%) and less severe in 3 patients (8.1%). The lowest eliciting dose, in terms of protein content, was 25 mg.

## Conclusion

OFC provides data regarding the allergic status and the eliciting dose in patients with a suspicion of peanut allergy. Caution is mandatory, since reactions following OFC may be more severe than the index reaction, despite the slow progression of doses.
